# Watt-level 10-gigahertz solid-state laser enabled by self-defocusing nonlinearities in an aperiodically poled crystal

**DOI:** 10.1038/s41467-017-01999-y

**Published:** 2017-11-22

**Authors:** A. S. Mayer, C. R. Phillips, U. Keller

**Affiliations:** Department of Physics, Institute of Quantum Electronics, ETH Zurich, 8093 Zurich, Switzerland

## Abstract

Femtosecond modelocked lasers with multi-gigahertz pulse repetition rates are attractive sources for all applications that require individually resolvable frequency comb lines or high sampling rates. However, the modelocked laser architectures demonstrated so far have several issues, including the need for single-mode pump lasers, limited output power, *Q*-switching instabilities and challenging cavity geometries. Here, we introduce a technique that solves these issues. In a two-dimensionally patterned quasi-phase-matching (QPM) device, we create a large, low-loss self-defocusing nonlinearity, which simultaneously provides SESAM-assisted soliton modelocking in the normal dispersion regime and suppresses *Q*-switching induced damage. We demonstrate femtosecond passive modelocking at 10-GHz pulse repetition rates from a simple straight laser cavity, directly pumped by a low-cost highly spatially multimode pump diode. The 10.6-GHz Yb:CaGdAlO_4_ (Yb:CALGO) laser delivers 166-fs pulses at 1.2 W of average output power. This enables a new class of femtosecond modelocked diode-pumped solid-state lasers with repetition rates at 10 GHz and beyond.

## Abstract (web summary)

Ultrafast lasers with multi-gigahertz repetition rates are desirable for applications requiring high sampling rates or resolvable frequency comb lines. Here, Mayer et al. use cascading of quadratic nonlinearities to passively modelock a femtosecond solid-state laser at a repetition rate of 10 GHz.

## Introduction

The advancement of passively modelocked solid-state lasers has provided the basis for the development of reliable low-noise frequency combs, which have nowadays become important tools for a large variety of metrology and spectroscopy applications. Frequency combs with line spacings above 10 GHz are powerful tools for spectroscopy with resolved comb lines^1^, the calibration of astronomical spectrographs^2–4^, arbitrary waveform generation^5^, terabit optical transmission systems^6^ and other applications that require easy access to the individual comb lines. Dual-combs in the gigahertz regime for spectroscopic applications with fast acquisition times have recently attracted the attention of the scientific community^7–10^. Furthermore, the ultrastable pulse train in the time domain can be used for low-noise microwave photonics^11^ and emerging optical computation systems based on coupled degenerate optical parametric oscillators^12–14^. Recently, pulse trains with multi-gigahertz repetition rates have also been used to generate dense pulse bursts that allowed micro-machining experiments to be performed in the ablation-cooled regime^15^.

To date, there are several approaches for generating frequency combs with large comb-line spacings such as electro-optic (EO) combs^16–18^, microresonator combs^19, 20^, Kerr lens modelocked (KLM) solid-state lasers^21, 22^ or comb-line filtering of e.g. modelocked fibre lasers in the few-hundred MHz-regime. There are different benefits and trade-offs for these techniques. Parametric comb generation using electro-optical modulators or microresonators offers a way to easily produce combs with >10 GHz line spacings. However, the formation of stable high-power femtosecond pulses is less straight-forward than with modelocked lasers^23^. EO combs, for example, require a series of nonlinear broadening and laser amplification stages, while microresonator combs require locking of a high-coherence parametric pump laser (single-spatial mode and single-optical frequency) to a high-Q resonator. On the other hand, in modelocked lasers the pulse train in the time domain and the spectral comb structure are instrinsically linked via Fourier transformation, and the intense output pulses are well suited to subsequent nonlinear frequency conversion. KLM modelocked solid-state lasers at 10-GHz or 15-GHz pulse repetition rates provide high-quality frequency combs, but the relatively low intracavity pulse energy at these high pulse repetition rates makes it more difficult to support KLM. The very tight focusing of the pump and laser beam required to initiate KLM via the gain medium requires an excellent pump beam quality (typically *M*^2^  ≈ 1)^21, 22^. Moreover, modelocking has to be initiated at the edge of the cavity stability region. As an alternative to GHz KLM lasers, a robust fibre-based oscillator with a repetition rate of a few 100 MHz and filtering the comb lines using Fabry–Pérot cavitites has also been sucessfully demonstrated as a way to reach comb-line spacings in the multi-gigahertz regime^24^. The complexity of such a system can however rapidly increase depending on the number of external cavities that need to be locked to achieve sufficient supression of the unwanted intermediate comb lines.

Semiconductor saturable absorber mirrors (SESAMs)^25^ enable robust stable passive soliton modelocking without any critical cavity stability criteria^26^. However, at multi-gigahertz repetition rates, *Q*-switching instabilities are a principal concern^27^. The high peak powers that occur during *Q*-switching instabilities damage the intracavity components before achieving sufficient power to exceed the threshold for stable continuous wave (cw) modelocking. To avoid these issues, V-shaped and Z-shaped cavities have been designed with properly placed curved mirrors to avoid focusing on critical cavity elements^28^. However, scaling these methods to the 10-GHz-regime has been elusive, since the small cavity size demands multi-functional optical components and severely limits their spatial arrangement.

A straight-cavity design with the SESAM as one of the end mirrors strongly relaxes those alignment constraints, but self-focusing of the beam and hence damage will occur in the cavity when operating in the conventional soliton modelocking regime. In this article, we introduce a new class of ultrafast lasers based on a simple straight cavity which solves the problems described above.

## Results

### Self-defocusing straight-cavity design

**Fig. 1:**
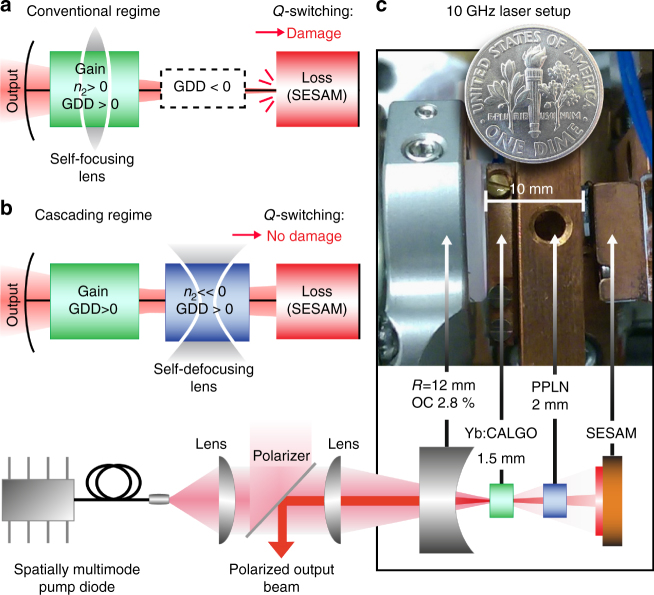
Modelocking regime and laser setup. **a** Conventional soliton modelocking, where positive self-phase modulation (SPM) with *n*_2_ > 0 is balanced by negative group-delay dispersion (GDD). In a straight-cavity configuration with a flat SESAM as an end-mirror, high peak powers during *Q*-switching instabilities cause self-focusing of the laser on the cavity elements, inducing damage and thereby preventing continuous wave modelocking. **b** Soliton modelocking using a self-defocusing nonlinearity to provide negative SPM that is balanced by positive GDD. In addition the strong negative effective Kerr nonlinearity creates a dynamic defocusing lens that leads to an increase of the mode size in the event of *Q*-switching instabilities, hence protecting the elements from damage. **c** Schematic and photo of the 10 GHz Yb:CALGO laser cavity, which is pumped by a spatially multimode pump diode (Methods)

Instead of relying on the intrinsic (weak) nonlinearity of the gain medium and negative dispersion compensating elements (conventional soliton modelocking regime, Fig. 1a), we engineer a device with a nonlinear refractive index (*n*_2,eff_) that is large and negative in sign, thus enabling femtosecond soliton modelocking at very low intracavity pulse energies and with net positive material dispersion (Fig. 1b).

Simultaneously, the negative *n*_2,eff_ implies a dynamic self-defocusing lens which suppresses damage: if an energetic pulse builds up due to *Q*-switching instabilities, the beam experiences strong self-defocusing, thus keeping the intensity below the damage threshold of the intracavity components.

**Fig. 2:**
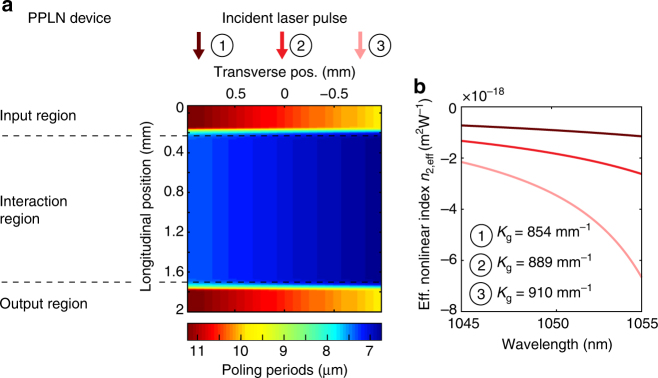
PPLN device. **a** Illustration showing a two-dimensional variation of the quasi-phase matching (QPM) domain structure. The grating vector *K*_g_ is varied smoothly along the *x*-direction to enable tuning of the nominal phase-mismatch and hence the effective nonlinear index *n*_2,eff_. The variation in *K*_g_ along the beam propagation direction has been designed in order to adiabatically turn the SHG interaction on/off: in the input region, the phase mismatch is adiabatically decreased to reach the value desired to obtain strong self-phase modulation (interaction region) and is then increased again in the output region to minimize the nonlinear losses. **b** Calculated effective nonlinear index in the device as a function of wavelength for three different values of the phase mismatch, corresponding to the transverse positions (1)–(3) with respect to the incoming beam. The calculations are based on material data for MgO-doped lithium niobate reported by Gayer et al.^49^

A compelling approach to obtain such a tuneable self-defocusing nonlinearity is via the cascading of quadratic nonlinearities (CQN)^29^. In CQN, a second-harmonic generation (SHG) crystal is used to generate a second-harmonic wave, which subsequently acts back upon the fundamental wave, thereby simulating a Kerr-like nonlinearity with an effective nonlinear refractive index *n*_2,eff_. Varying the phase-mismatch of the SHG process allows the sign and magnitude of *n*_2,eff_ to be engineered, which has enabled soliton modelocking in the normal dispersion regime^30–35^. A potential drawback of the CQN technique are the losses associated to residual SHG. The losses one obtains using conventional CQN, e.g. in birefringent crystals, can easily exceed 0.5%^34^ which is intolerable for a SESAM-modelocked laser in the multi-gigahertz regime. The solution comes in the form of quasi-phase matching (QPM) materials: lithographic patterning of such materials now allows us to engineer a large, self-defocusing nonlinearity that is adiabatically excited via a non-uniform QPM structure in order to suppress the SHG losses by more than an order of magnitude (Methods). The device used here consists of a 2-mm-long periodically poled lithium niobate crystal (PPLN) that is two-dimensionally patterned (Fig. 2). In earlier work^33^, we have presented the operating principles of this type of device, whose functionality has now for the first time been leveraged to enable a new type of multi-gigahertz all-self-defocusing straight cavity.

**Fig. 3:**
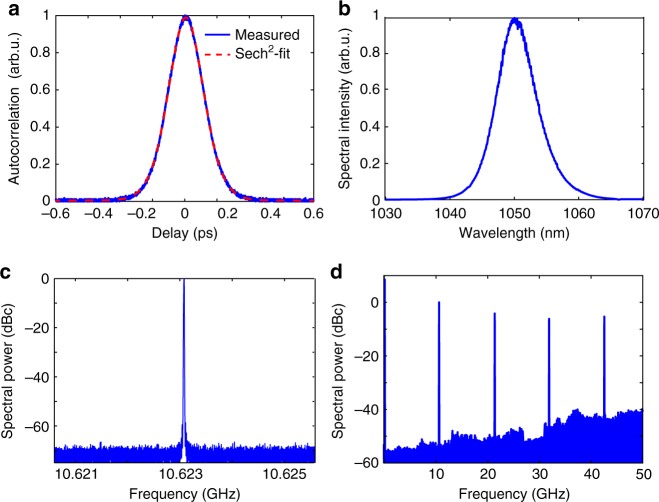
Modelocking characterization of the 10-GHz Yb:CALGO laser at 1.2 W of average output power. **a** Intensity autocorrelation of the transform-limited 166 fs pulses. **b** Optical spectrum centred at 1050 nm with a bandwidth of 7 nm (full width at half maximum, FWHM) measured with a resolution bandwidth (RBW) of 0.08 nm. **c** 5-MHz span microwave spectrum recorded with an RBW of 3 kHz showing the repetition rate signal at 10.62 GHz **d** 50-GHz span microwave spectrum recorded with an RBW of 100 kHz

**Fig. 4:**
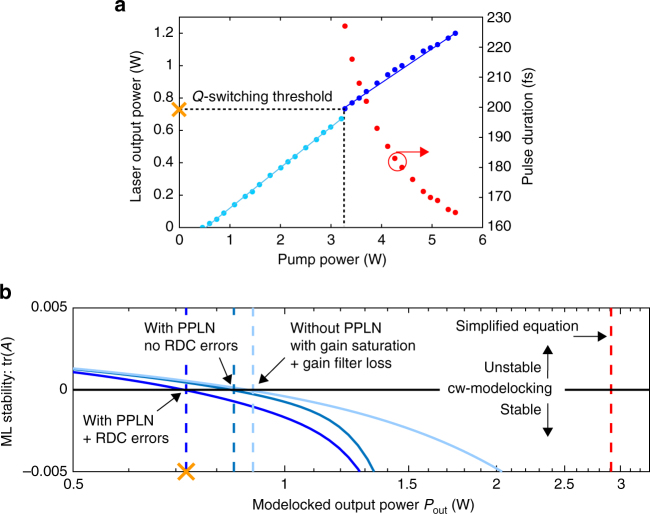
Laser operation regimes. **a** Experimentally measured output power and pulse duration vs. diode pump power. The slope efficiency amounts to 25% in the Q-switching region (light blue) and 21% in continuous wave (cw) modelocking (dark blue). **b** *Q*-switching threshold of the 10 GHz laser assuming a 2.8% output coupler: The modelocking stability is predicted via a matrix *A* (Methods), which describes the linearized dynamics of the laser gain and pulse energy around the steady state. For cw modelocking, trace(*A*) < 0 is required^46^. The blue dashed lines represent crossing points where trace(*A*) = 0 under the following assumptions. Right: taking first only the effect of gain saturation and gain filtering into account; middle: adding the effect of a perfect QPM structure; left: assuming a realistic QPM structure with random duty cycle (RDC) errors in the ferroelectric domains arising during fabrication^48^. Including these RDC errors, we obtain a predicted *Q*-switching threshold output power of 724 mW which is remarkably close to the experimentally observed value of 734 mW. For comparison, the red dashed line shows the value predicted by a widely used analytical model^39^

Values for the effective nonlinear index *n*_2,eff_ ranging from −1 × 10^−18^ to −7 × 10^−18^ m^2^W^−1^ can be achieved in this PPLN device, which is approximately two orders of magnitude higher than the intrinsic third-order material nonlinearities of conventional wide band-gap materials. For example, the nonlinear index of the gain material Yb:CALGO^36^ amounts only to *n*_2,intrinsic_^CALGO^ ≈ + 8 × 10^−20^ m^2^W^−1^. The best modelocking results (presented in Figs. 3 and 4) were obtained at transverse position 2, indicated in Fig. 2a. This position corresponds to a grating vector *K*_g_ = 889 mm^−1^, leading to a phase mismatch of 44.9 mm^−1^ at 1052 nm. The net total intracavity GDD amounts to ≈ + 1280 fs^2^ per roundtrip (material dispersion of the 2-mm-long PPLN and the 1.5-mm-long Yb:CALGO crystal, the SESAM was measured to have a reasonably flat GDD profile with a negligible contribution of −66 fs^2^ at 1050 nm).

### Modelocking results

The lasing operation can be divided into two regions: at low pump power, mode-beating and *Q*-switching instabilities are observed. When increasing the pump power further, the laser reliably transitions into self-starting soliton modelocking at around 734 mW average output power (Fig. 4a) initiated and stabilized by the SESAM. Stable cw modelocking with a TEM_00_ beam profile and an *M*^2^ = 1.01 (measured with the Thorlabs BP104-IR beam profiler) is maintained up to an average power of 1.2 W. At this power, the laser can be operated for several hours a day without noticeable degradation of the modelocking parameters and repeatable performance over several months has been observed. A frequency-dependent amplitude noise measurement is shown in Supplementary Fig. 1 (and discussed in Supplementary Note 1). If the pump power is further increased, higher order spatial modes start to lase, which become visible in the laser beam profile and appear as sidepeaks in the radio-frequency trace. The onset of higher order modes can be explained by the strong beam divergence of the multimode pump within the length of the gain medium, which allows for non-TEM_00_-modes to experience sufficient gain above a certain pump power. Using a pump with a lower *M*^2^-value and/or a shorter gain crystal would mitigate this effect. Over this range, the duration of the transform-limited pulses decreases from 227 to 166 fs, consistent with the prediction of soliton modelocking, and the centre wavelength shifts from 1052 to 1050 nm. Shorter pulse durations could be achieved by decreasing the net positive intracavity dispersion, i.e. by using a Gires–Tournois interferometer-type coating on the output coupler.

### Laser cavity dynamics

To understand the threshold for cw modelocking, the influence of soliton shaping and gain filtering, as well as the role of the PPLN device on the *Q*-switching threshold has been quantified using numerical simulations (Fig. 4b). These results show that the PPLN device helps to reduce the *Q*-switching threshold (Methods).

**Fig. 5:**
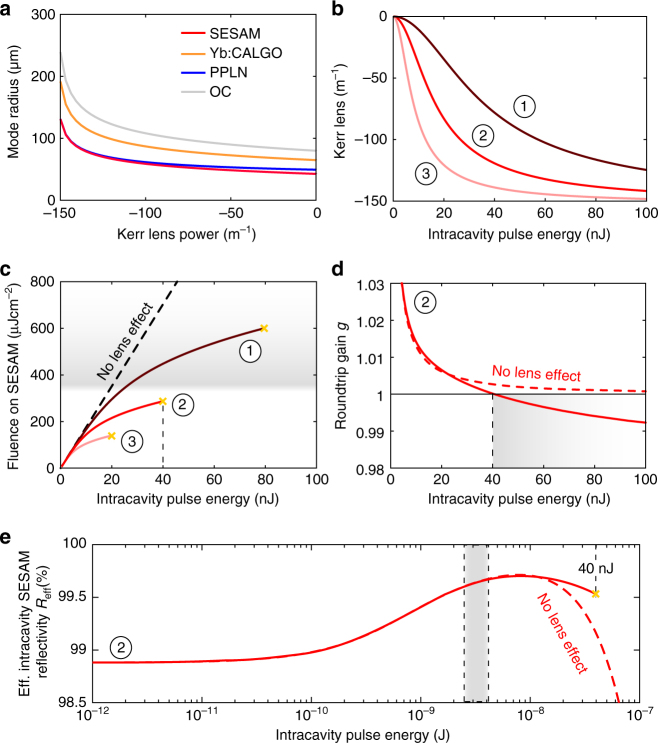
Effect of the dynamic defocusing lens in the PPLN on various parameters. **a** Intracavity mode radius as function of PPLN Kerr lens power. **b** PPLN Kerr lens as function of intracavity pulse energy for the three different PPLN grating vector values *K*_g_ of (1) 854 mm^−1^, (2) 889 mm^−1^ and (3) 910 mm^−1^. At a wavelength of 1052 nm, these values correspond to effective nonlinear indices *n*_2,eff_ of (1) −0.99 × 10^−18^ m^2^W^−1^, (2) −2.07 × 10^−18^ m^2^W^−1^ and (3) −4.24 × 10^−18^ m^2^W^−1^, respectively **c** Fluence on the SESAM with and without the effect of the defocusing lens. The grey shaded area corresponds to the region where damage of the SESAM is likely to occur. **d** Gain *g* (ratio of input intensity to output intensity after two passes through the gain crystal) as a function of intracavity pulse energy. Solid line: with lens effect; dashed line: assuming a constant mode size in the Yb:CALGO. Above a certain intracavity pulse energy (grey area), no more gain is experienced by the pulses. **e** Effective intracavity SESAM reflectivity for PPLN position (2): for each pulse energy, the corresponding change in mode size and pulse duration is taken into account. The experimentally observed cw modelocking region from 2.4 to 4 nJ is shaded in grey

However, a low *Q*-switching threshold alone is not sufficient to guarantee successful modelocking. Damage may still occur due to *Q*-switching instabilities that inevitably build up inside the cavity while ramping up the pump power. By exploiting dynamical self-defocusing effects, we are able to suppress damage while transitioning through this regime. In Fig. 5a we show the mode radius on all the cavity elements as a function of the negative Kerr lens, which is induced in the PPLN device. The Kerr lens power *F* can be expressed as a function of the pulse energy *E*_p_, the corresponding soliton pulse duration *τ*_FWHM_, the mode radius in the PPLN *w*_PPLN_ and the length of the PPLN crystal *L*_PPLN_^37^:

1 $$F = 1.76\frac{{n_{{\mathrm{2,eff}}}L_{{\mathrm{PPLN}}}E_{\mathrm{p}}}}{{\pi w_{{\mathrm{PPLN}}}^4\tau _{{\mathrm{FWHM}}}}}$$

This defocusing lens depends on *n*_2,eff_ (Fig. 5b), and thus on the position of the PPLN as described in Fig. 2. As a result of this dynamic defocusing Kerr lens, the fluence on the SESAM does not increase linearly anymore, but instead shows a clamping behaviour as the mode size increases (Fig. 5c). Moreover, the laser mode size in the gain crystal also increases with stronger Kerr lensing in the PPLN, and hence for a fixed pump power and pump mode size, the gain will eventually drop below unity at a certain pulse energy (Fig. 5d). Based on this energy, we estimate the maximum fluence that may occur during *Q*-switching (yellow crosses in Fig. 5). For the experimental modelocking parameters (PPLN at position 2) and for a pump power corresponding to the *Q*-switching threshold, we obtain an upper limit of ~40 nJ, beyond which the pulse energy is clamped (the same calculation was performed to obtain the clamping points in Fig. 5c for the other values of *n*_2,eff_). Next we evaluate the response of the SESAM. The AlAs-embedded single-InGaAs-quantum well SESAM used has a saturation fluence of *F*_sat_ ≈ 8 µJ cm^−2^, a modulation depth Δ*R* ≈ 1%, non-saturable losses ≈ 0.12% and an inverse saturable absorption coefficient *F*_2_ ≈ 500 mJ cm^−2^. These parameters were extracted from fitting the SESAM reflectivity curve for a Gaussian beam^38^ to the reflectivity values measured at a fixed 170-fs pulse duration and constant spot size on the SESAM. To mimic the real response of the SESAM inside the 10-GHz cavity, we need to incorporate the change in mode size due to the PPLN Kerr lens for each energy point. Additionally, since the pulse duration of the soliton decreases with energy, the pulse duration-dependent *F*_2_ coefficient^39^ needs to be scaled for each energy value as well. This leads to the effective intracavity SESAM reflectivity curve shown in Fig. 5e. This curve shows how the lens effect prevents operation too far into the rollover of the reflectivity, which in turn prevents strong absorption in the SESAM and thus potential damage.

## Discussion

In conclusion, by combining SESAM modelocking with adiabatic excitation of cascaded quadratic nonlinearities, we overcome the issues faced by conventional femtosecond modelocked lasers that have strongly limited access to 10-GHz repetition rates until now. We demonstrated a straight-cavity 10-GHz SESAM-modelocked laser that operates in the positive GDD regime. The intracavity multi-functional 2D-QPM PPLN crystal shapes the pulses in time by providing negative SPM for soliton formation while minimizing the intracavity loss, and simultaneously acts as a dynamic self-defocusing lens, which protects the cavity elements from *Q*-switching related damage. Combining these effects allowed us to achieve for the first time femtosecond Watt-level fundamental modelocking of a diode-pumped solid-state laser with a repetition rate above 10 GHz. The SESAM as well as the PPLN device are fabricated on a wafer scale, with the custom QPM pattern on the latter defined by a lithographic mask. The pulsed nature of this 1-µm laser source makes it well suited to various frequency conversion techniques with novel nonlinear platforms, such as low-energy supercontinuum generation in silicon nitride waveguides^40^ or high-gain optical parametric amplification^41^ to reach the mid-infrared spectral region. The modelocking technique based on self-defocusing nonlinearities can furthermore be deployed at other wavelengths and is expected to be especially favourable at longer wavelengths due to the reduced group velocity mismatch between the fundamental and the second harmonic in the PPLN crystal. The new class of ultrafast lasers enabled by this technique represents an important step towards compact high-power optical frequency combs beyond 10-GHz.

## Methods

### Laser cavity details

The 1.5-mm-long Yb:CALGO gain crystal is pumped by an internally wavelength-stabilized, spatially multimode diode (Lissotschenko Mikrooptik GmbH, *M*^2^ ≈ 36) capable of providing up to 60 W at 980 nm. The pump beam is focused through a 12-mm-radius pump-transparent mirror that acts as a 2.8% output coupler for the 1050 nm laser centre wavelength. In order to minimize the thermal load inside the laser cavity, the vertically polarized pump light, which would only be weakly absorbed in the gain crystal, is removed using a polarizing beam splitter. The beam splitter at the same time allows the vertically polarized laser beam to exit.

### PPLN device operating principle

The principle behind the self-defocusing nonlinearity is phase-mismatched SHG, often referred to as CQN. Far from phase-matching, the fundamental experiences an effective third-order nonlinear response that can be expressed as a nonlinear index contribution *n*_2,CQN_ tunable via the phase-mismatch ∆*k*. Adding it to the intrinsic material nonlinear index *n*_2,intr_, yields the total effective nonlinear index *n*_2,eff_:

2 $$n_{2,{\mathrm{eff}}} = n_{2,{\mathrm{intr}}} + n_{{\mathrm{2,CQN}}} = n_{2,{\mathrm{intr}}} - \frac{{4\pi d_{{\mathrm{eff}}}^2}}{{\epsilon _0cn_{\mathrm{F}}^2n_{{\mathrm{SH}}}\lambda _{\mathrm{F}}}}\frac{1}{{\Delta k}}$$

(*λ*_F_: wavelength of the fundamental, *n*_F/SH_: linear refractive index at the fundamental/second harmonic, *d*_eff_: effective nonlinear coefficient).

In our PPLN device, the QPM periods vary transversely across the chip. By translating this 2D-QPM PPLN device with respect to the incident laser beam, a tunable phase mismatch $\Delta k(x) = k_{{\mathrm{SH}}} - 2k_{\mathrm{F}} - K_{\mathrm{g}}(x)$ can be obtained, where $k_{{\mathrm{F/SH}}} = 2\pi n_{{\mathrm{F/SH}}}/\lambda _{{\mathrm{F/SH}}}$ denotes the wave vector of the fundamental wave (F) and its second harmonic (SH) respectively.

In order to obtain the largest magnitude of *n*_2,eff_, the phase mismatch Δ*k* has to be made as small possible within the validity of the CQN regime. However, in a normal CQN device, the fraction of power lost to SHG increases according to $\alpha \sim 1/(\Delta k)^2$. Such losses resemble an inverse saturable absorption in the laser, limiting the nonlinearity available before modelocked operation is destabilized. This issue is especially critical at high repetition rates, where large nonlinearities are required due to the low intracavity pulse energy.

We have developed a new technique to suppress these losses: adiabatic excitation of quadratic solitons^33^. In this technique, the QPM period is rapidly but smoothly moved far from phase-matching at the input and output sides of the device. In this way, the second-harmonic light, which mediates the large effective nonlinear refractive index, is adiabatically switched “on” via the input segment, is large in the middle segment (leading to a large and negative *n*_2,eff_ in that segment), and is switched “off” via the output segment, as illustrated in Fig. 2 of the main text. With this technique, the resulting nonlinear losses can be decreased by an order of magnitude or more compared to a non-apodized device^33^. The nonlinear chirp QPM profiles used are analogous to apodization in chirped QPM devices^42, 43^, where they efficiently excite adiabatic three-wave mixing processes^44^.

### PPLN operating point

In our PPLN device, the value of the grating *k*-vector *K*_g_ in the interaction region (as indicated in Fig. 2a) varies continuously across the transverse position in the device (i.e. perpendicular to the beam propagation direction). This value varies from 854 to 934 mm^−1^ across the transverse profile of the device. The three example operating points depicted in Fig. 2a and the corresponding values of *n*_*2*,eff_ in Fig. 2b illustrate the trade-offs involved in optimizing modelocking performance. When operating closer to position (1) (large phase-mismatch, *K*_g_ = 854 mm^−1^), the self-defocusing lens effect becomes too weak and *Q*-switching damage can be observed (Fig. 5c). When moving towards a too-small phase mismatch (position (3), *K*_g_ = 910 mm^−1^), the nonlinear losses increase and are accompanied by a self frequency shift effect^45^. These effects cause the laser to react by shifting to a shorter centre wavelength, which however at the same time moves the pulse away from the spectral region with highest gain in the Yb:CALGO crystal. Optimum modelocking performance was obtained between these extrema, with the PPLN device placed in position (2) (*K*_g_ = 889 mm^−1^).

### Numerical prediction of the *Q*-switching threshold

In a general sense, stable cw modelocking can be characterized as a state where small deviations around the steady-state gain *g*_s_ and pulse energy *E*_s_ will be damped by the system, instead of being amplified as it is the case for *Q*-switching instabilities. Mathematically, these linearized equations for pulse energy *E* and gain *g* can be expressed in vector form^46^,

3 $$T_{\mathrm{R}}\frac{{\mathrm{d}}}{{{\mathrm{d}}T}}\left( {\begin{array}{*{20}{c}} {\Delta E} \\ {\Delta g} \end{array}} \right) = A\left( {\begin{array}{*{20}{c}} {\Delta E} \\ {\Delta g} \end{array}} \right),\quad A = \left( {\begin{array}{*{20}{c}} {\frac{{\partial G}}{{\partial E}}E} & {\frac{{\partial G}}{{\partial g}}E} \\ { - g\frac{1}{{E_{{\mathrm{sat,L}}}}}} & { - \left( {\frac{{T_{\mathrm{R}}}}{{\tau _{\mathrm{L}}}} + \frac{E}{{E_{{\mathrm{sat,L}}}}}} \right)} \end{array}} \right)$$

where *T*_R_ denotes the cavity round-trip time, *E*_sat,L_ the saturation energy of the laser gain medium, *τ*_L_ is the upper-state lifetime of the gain medium (420 µs for Yb:CALGO^36^) and *G* corresponds to the total gain (sum of all cavity gain and loss terms), which is zero for the steady state. For stability, the trace of this matrix needs to be negative^46^. The *Q*-switching threshold can thus be defined as the pulse energy *E*_th_ for which trace(*A*) = 0. In cases where the only saturable losses arise from the SESAM, gain filtering is neglected and the laser is operating many times above threshold, i.e. $(E\tau _{\mathrm{L}})/(E_{{\mathrm{L,sat}}}T_{\mathrm{R}}) > > 1$, the *Q*-switching threshold condition above leads to the following (often cited) simplified equation^39^:

4 $$E_{{\mathrm{th}}}^2 = \frac{{E_{{\mathrm{sat,A}}}\Delta R}}{{\frac{1}{{E_{{\mathrm{sat,L}}}}} + \frac{1}{{A_{\mathrm{A}}F_2}}}}$$

(*E*_sat,A_: saturation energy of the SESAM, *A*_A_: beam area on SESAM, *F*_2_: inverse saturable absorption coefficient, Δ*R*: modulation depth). Evaluating this expression for the parameters of this 10-GHz laser yields a predicted intracavity pulse energy threshold of 9.9 nJ, corresponding to an average output power of 2.9 W (red dashed line in Fig. 4), which is about 4 times higher than we experimentally observe. Although Eq. 4 may be convenient to use, it does not provide the full picture.

We have developed a complete calculation of trace(*A*) in the steady-state for quasi-three-level modelocked lasers. We include soliton shaping, gain filtering using directly measured cross section data for Yb:CALGO^47^, as well as the exact SESAM and PPLN responses, accounting for the transverse beam profile of the laser and pump beams in each intracavity component (a flat-top pump beam is assumed for simplicity).

Neglecting the PPLN crystal losses, this precise calculation already reduces the predicted threshold to an average power of ~900 mW. To further improve the accuracy, the total gain *G* needs to contain the residual nonlinear SHG losses in the PPLN crystal, which are analogous to inverse saturable absorption effects. Although the nonlinear losses are minimized by QPM design as described above, we need to assume random duty cycle (RDC) errors due to imperfect fabrication^48^. Assuming a realistic QPM period jitter of 0.2 µm increases the nonlinear losses from ideally 0.01 to 0.05%. Including these RDC errors, the predicted *Q*-switching threshold output power drops to 724 mW, which is in very good agreement with the experimentally observed threshold.

### Data availability

The data and simulations codes that support the findings of this study are available from the corresponding authors upon request.

## Electronic supplementary material

Supplementary Information

Peer Review File
