# Author Correction: Watt-level 10-gigahertz solid-state laser enabled by self-defocusing nonlinearities in an aperiodically poled crystal

**DOI:** 10.1038/s41467-018-03816-6

**Published:** 2018-04-11

**Authors:** A. S. Mayer, C. R. Phillips, U. Keller

**Affiliations:** Department of Physics, Institute of Quantum Electronics, ETH Zurich, 8093 Zurich, Switzerland

Correction to: *Nature Communications* 10.1038/s41467-017-01999-y, published online 22 November 2017

The original version of this Article contained an error in Fig. 3a, in which the delay axis was incorrectly labeled in increments of 0.5 ps instead of the correct 0.2 ps. This has been corrected in both the PDF and HTML versions of the Article.

