# Supplementary material for: Watt-level 10-gigahertz solid-state laser enabled by self-defocusing nonlinearities in an aperiodically poled crystal
Source: Nat Commun. 2017 Nov 22;8:1673. doi: 10.1038/s41467-017-01999-y (PMC5698476; doi:10.1038/s41467-017-01999-y)
Supplement: Supplementary file 1 — Supplementary Information [file 41467_2017_1999_MOESM1_ESM.pdf]

Supplementary Note 1

The amplitude noise during clean cw modelocking is very similar to the noise measured in our SESAM modelocked 1-GHz Yb:CALGO laser[1], which operated in the conventional negative-dispersion soliton regime, and did not contain a PPLN crystal. Hence, the cascaded quadratic nonlinearities (CQN) modelocking regime we have accessed during this work does not seem to alter the amplitude noise in any significant way. Both lasers are pumped with the same multimode pump diode, which – as can be seen in Fig. S1 - is the limiting factor for the noise of both lasers. However, by providing feedback to the pump current, we have shown in Klenner et al.[1] that this noise can be handled, leading to a tight lock of the offset frequency of the 1-GHz laser. At 10 GHz, a broader feedback bandwidth is expected to be needed due to the scaling of the phase noise with the repetition rate, but we expect that using suitable electronics, this should still be feasible. Stabilization of the repetition rate frequency could furthermore be implemented by mounting the SESAM on a piezo-element to stabilize the length of the cavity as demonstrated recently by Hakobyan et al.[2].

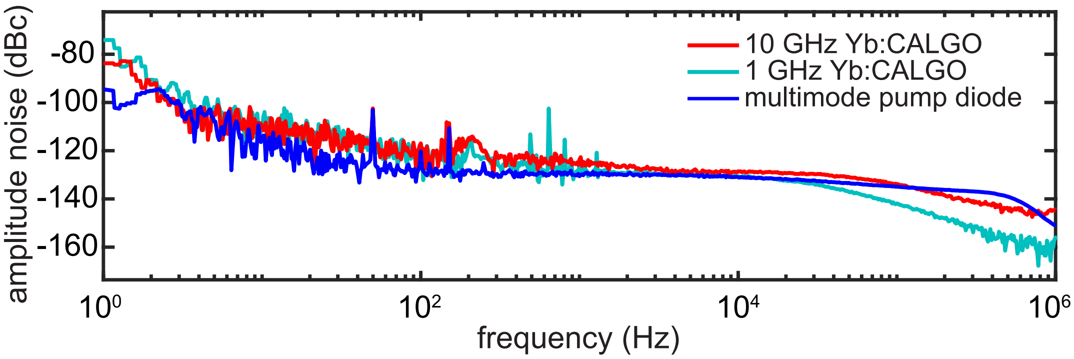

Supplementary Figure 1| Amplitude noise comparison. Amplitude noise of the 10 GHz laser (red) vs. the 1 GHz Yb:CALGO laser (turquoise) and the spatially multimode pump diode (blue). The root-mean-square (rms) noise integrated over the interval (1 Hz, 1 MHz) amounts to: 10 GHz: 0.019 %, 1 GHz: 0.010 %, pump: 0.017 %

Supplementary References

1. Klenner A., Mayer A.S., Johnson A.R., Luke K., Lamont M.R.E., *et al.* Gigahertz frequency comb offset stabilization based on supercontinuum generation in silicon nitride waveguides. *Opt. Express* **24,** 11043-11053 (2016).
2. Hakobyan S., Wittwer V.J., Brochard P., Gürel K., Schilt S., *et al.* Full stabilization and characterization of an optical frequency comb from a diode-pumped solid-state laser with GHz repetition rate. *Opt. Express* **25,** 20437-20453 (2017).
