# Supplementary material for: Watt-level 10-gigahertz solid-state laser enabled by self-defocusing nonlinearities in an aperiodically poled crystal
Source: Nat Commun. 2017 Nov 22;8:1673. doi: 10.1038/s41467-017-01999-y (PMC5698476; doi:10.1038/s41467-017-01999-y)
Supplement: Supplementary file 2 — Peer Review File [file 41467_2017_1999_MOESM2_ESM.pdf]

Editorial Note: this manuscript has been previously reviewed at another journal that is not operating a transparent peer review scheme. This document only contains reviewer comments and rebuttal letters for versions considered at Nature Communications.

REVIEWERS' COMMENTS:

Reviewer #1 (Remarks to the Author):

The authors have answered my concerns and suggestions in previous review. I recommend accepting the manuscript for publication in Nature Communications.

Reviewer #2 (Remarks to the Author):

The authors present a novel approach to mode lock a 10 GHz solid state laser by using a self-defocusing nonlinearity in a cascaded chi-2 material. The techniques are state-of-the-art and the implementation is scientifically sound. The generation of mode-locked lasers and frequency combs at 10+ GHz is a very active area of research that is attracting significant attention from a broad range of scientists. Thus, I believe the paper will be interesting and impactful to a broad audience.

The authors have done an excellent job responding to the comments raised in a earlier round of reviewing and I think the paper is in good condition to now be published.

Response to the Reviewer Comments

We thank the reviewers for their helpful and fair feedback in the previous review

round and for their positive final judgment on our revised manuscript.
